# GEOM-drugs revisited: toward more chemically accurate benchmarks for 3D molecule generation

**DOI:** 10.1039/d5dd00206k

**Published:** 2025-10-02

**Authors:** Filipp Nikitin, Ian Dunn, David Ryan Koes, Olexandr Isayev

**Affiliations:** a Ray and Stephanie Lane Computational Biology Department, Carnegie Mellon University Pittsburgh PA USA olexandr@olexandrisayev.com; b Department of Chemistry, Carnegie Mellon University Pittsburgh PA USA; c Department of Computational and Systems Biology, University of Pittsburgh Pittsburgh PA USA

## Abstract

Deep generative models have shown significant promise in generating valid 3D molecular structures, with the GEOM-drugs dataset serving as a key benchmark. However, current evaluation protocols suffer from critical flaws, including incorrect valency definitions, bugs in bond order calculations, and reliance on force fields inconsistent with the reference data. In this work, we revisit GEOM-drugs and propose a corrected evaluation framework: we identify and fix issues in data preprocessing, construct chemically accurate valency tables, and introduce a GFN2-xTB-based geometry and energy benchmark. We retrain and re-evaluate several leading models under this framework, providing updated performance metrics and practical recommendations for future benchmarking. Our results underscore the need for chemically rigorous evaluation practices in 3D molecular generation. Our recommended evaluation methods and GEOM-drugs processing scripts are available at https://github.com/isayevlab/geom-drugs-3dgen-evaluation.

## Abstract 2

Revisiting GEOM drugs: corrected metrics and novel energy-based structural benchmark enable rigorous evaluation of 3D molecule generative models.

## Graphical abstract

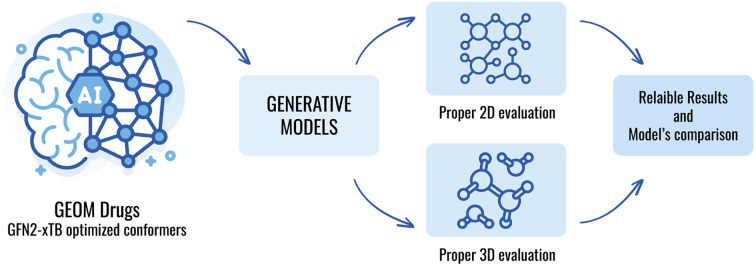

## Introduction

Generative models for molecules are an emerging paradigm that enables the construction of novel molecules in 2D or 3D.^1,2^ These AI models learn the patterns and distribution of existing molecular data to generate previously unseen chemical structures. By encoding molecular information into mathematical representations and then sampling from a learned distribution, these models facilitate efficient exploration of vast chemical space. The field continues to evolve rapidly and is not yet mature.

The field of cheminformatics has established fundamental protocols^3,4^ and best practices^5,6^ for achieving ML models with high statistical rigor and external predictive power.^4^ Here, critical steps such as data preparation, chemical structure curation, outlier detection, dataset balancing, and rigorous ML model validation must be included into the overall data workflow. Multiple studies emphasized that chemical structure curation should be treated as a separate and critical component of any cheminformatics research.^6^ Seminal studies showed that accumulated errors and incorrect processing of chemical structures can significantly reduce the accuracy of ML models.^7^

The GEOM data set^8^ is one of the most widely used large-scale high-accuracy datasets of molecular conformations. A subset of GEOM containing drug-like molecules, known as GEOM-drugs, has become a foundational benchmark for developing 3D molecular generative models. The frequent use of GEOM-drugs in this field has given rise to a somewhat standardized set of metrics to evaluate the quality of generative models trained on this dataset. In this work, we identify several critical issues in how state-of-the-art 3D molecular generative models are evaluated. We believe these issues mislead the research community and limit progress in the field.

First, we highlight three major problems with the commonly used “molecular stability” metric, which measures whether atoms have valid valencies. One of the original implementations contained a bug. This bug caused chemically implausible valencies to be counted as valid, which inflated stability scores. This flawed implementation was reused by several follow-up works,^9–14^ resulting in a significant body of work with misleading characterizations of model performance.

Second, many recent works lack rigorous and chemically grounded evaluation of 3D structures, which continues to hinder progress in generative modeling. Common issues include the use of oversimplified atom–atom distance lookup tables to evaluate the validity of generated 3D structures,^15–20^ reliance on distribution-based metrics that are difficult to interpret,^10,14^ and the use of energy evaluations at different levels of theory than the training data.^9,21^

To address these issues, this paper provides:

(1) A refined dataset split of GEOM-drugs, which excludes molecules where GFN2-xTB calculations fractured the original molecule.

(2) An updated molecule stability metric with a chemically accurate valency lookup table that is derived from this refined dataset.

(3) An energy-based evaluation methodology for an accurate and chemically interpretable assessment of generated molecular 3D geometries.

We retrained several widely used generative models on our reprocessed dataset and updated the evaluation metrics to address previously observed issues. The relative rankings of the models remained largely consistent. However, our updates yielded practical improvements. These improvements highlight the importance of rigorous and accurate evaluation practices in the field.

## Revisiting the molecule stability metric

Valency in chemistry refers to the combining capacity of an atom or element, describing how many chemical bonds it can form with other atoms. It is defined as the sum of bond orders of its covalent bonds. Due to chemical constraints (*e.g.*, the octet rule), atoms of a given element and formal charge typically exhibit only a few plausible valencies; for instance, neutral carbon almost exclusively has a valency of 4. Molecules violating these valency constraints are chemically unstable. Thus, generative models must produce molecules adhering to these rules. A practical evaluation of generative models involves measuring the fraction of atoms with valid valencies. This evaluation was originally proposed by Hoogeboom *et al.*,^17^ a seminal work applying diffusion models to *de novo* molecule generation; this metric is known as “atom stability”. The metric “molecule stability” is defined as the fraction of molecules where all atoms have valid valencies. A valid valency is defined as one observed in the training data. A “lookup table” of valid valencies, consisting of tuples of (element, formal charge, valency), is created from the training set.

**Fig. 1:**
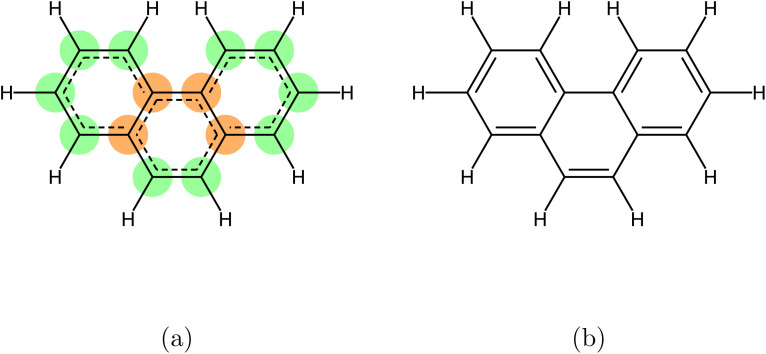
An example of a molecule where the assumption that aromatic bonds contribute 1.5 to atomic valency holds only partially. In the aromatic form of triphenylene (a), the green-highlighted atoms are correctly classified as stable under the 1.5 assumption, while others are misclassified. In contrast, the kekulized representation (b) resolves the ambiguity and yields chemically accurate valency assignments across all atoms. This illustrates the limitations of the 1.5 approximation in polycyclic aromatic systems.

Valency can be computed as the sum of bond orders in a molecule's kekulized form,‡‡Kekulization is the process of generating an alternative structure for a molecule where aromatic bonds are converted to alternating single and double bonds. where bonds are explicitly represented as single, double, or triple. This approach works reliably for molecules without aromatic bonds. When aromatic bonds are introduced, however, valency computation becomes more complex. In simple cases such as benzene, one can assume each aromatic bond contributes 1.5 to the valency, yielding the correct total (*e.g.*, carbon atoms in benzene are correctly assigned a valency of 4). But in more complex aromatic systems, this assumption may not hold, and valency contributions can vary depending on the bonding environment and resonance structures (see Fig. 1).

The authors of Hoogeboom *et al.*^17^ proposed atom and molecule stability metrics to evaluate the correctness of the raw output of generative models. They noted that traditional validity metrics, defined as the fraction of molecules that can be sanitized with RDKit, can be misleading, as RDKit may implicitly adjust hydrogen counts or modify aromaticity, altering the predicted molecule. We generally support the idea of assessing raw valencies, especially for models that explicitly generate both atoms and bonds because it provides a more chemically grounded evaluation. Unlike validity, stability captures whether the generated molecules respect elemental valence constraints without relying on post-processing. However, we identified that early implementations of the molecular stability generally contain flaws related to the aforementioned complications of counting valencies in aromatic systems.

### Critical flaws in existing molecule stability evaluation

**Fig. 2:**
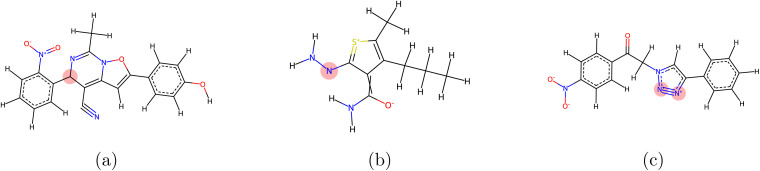
Examples of molecules that pass the molecular stability evaluation under commonly used criteria. These flawed metrics erroneously classify chemically invalid configurations as stable. (a) contains a neutral carbon with three single bonds. (b) Contains a neutral nitrogen with two single bonds. (c) Contains a nitrogen atom with +1 charge bonded *via* both a triple bond and an aromatic bond. In each subfigure the mentioned invalid valency is highlighted with a red circle.

We identify multiple critical issues with the valency evaluation methods used in popular molecular generative models. The issues we identify obscure instances where generative models produce chemically implausible structures. One of the pioneering models, MiDi,^14^ implemented a valency calculation method in which the valency contributions for all aromatic bonds were rounded to 1 instead of the intended value of 1.5. Thus, the valency computation for most atoms participating in aromatic bonds is incorrect. More importantly, it appears that the flawed valency computation was also used to construct the valency lookup table that is used to classify generated atoms as “stable” or not, resulting in a lookup table with chemically implausible entries. For instance, the lookup table allows for neutral carbon with a valency of 3 and neutral nitrogen with a valency of 2. Implausible entries in the valency lookup table mask failures of the generative model and produce artificially inflated molecular stability values. Due to widespread reuse of MiDi's code, this numerical error propagated to several works including EQGAT-Diff,^10^ SemlaFlow,^9^ Megalodon,^13^ and FlowMol.^11,12^ Other models, such as JODO^15^ and NextMol,^22^ computed valencies using an alternative approach based on RDKit kekulization. However, they still relied on an inappropriate lookup table for defining valid valency ranges (Fig. 2).

In their current form, widely-used molecular stability metrics may not provide accurate representations of model performance. The implementation of these metrics must be corrected to enable future progress in the development of *de novo* generative models.

### A chemically grounded solution of molecule stability metric

Two key solutions are necessary to correct the aforementioned problems with the molecular stability metric: fixing the valency computation bug for aromatic bonds and recomputing the valency lookup table. We quantify the effects of our proposed solutions by re-evaluating models that used the faulty molecular stability metric in their original publications: EQGAT-Diff,^10^ Megalodon-quick,^13^ SemlaFlow,^9^ FlowMol2,^12^ and Megalodon-flow.^13^ The results of these reevaluations are shown in Table 1. All metrics were computed using 5000 generated molecules per model.

**Table 1:** Comparison of molecular stability (MS) and connected validity (V&C) across models and processing pipelines. The left section reports results obtained using the original GEOM-drugs dataset and evaluation code: “original” denotes the values from metric implementations published in prior work, “1.5 Arom” reflects scores if aromatic bonds contribute 1.5 to valency, and “Arom-dependent valence” shows scores based on valency computed as (*n*_arom_, *v*_other_). The right section presents results obtained by retraining on fully kekulized molecules. V&C (valid & connected) refers to the fraction of molecules that are both chemically valid and consist of a single connected component

Model	MS original	MS 1.5 Arom	MS Arom-dependent valence	V&C	MS	V&C
EQGAT^10^	0.935 ± 0.007	0.451 ± 0.006	0.899 ± 0.007	0.834 ± 0.009	0.878 ± 0.007	0.891 ± 0.010
JODO^15^	0.981 ± 0.001	0.517 ± 0.012	0.963 ± 0.005	0.879 ± 0.003	0.940 ± 0.003^a^	0.923 ± 0.004^a^
Megalodon-quick^13^	0.961 ± 0.003	0.496 ± 0.017	0.944 ± 0.003	0.900 ± 0.007	0.957 ± 0.006	0.962 ± 0.005
SemlaFlow^9^	0.980 ± 0.012	0.608 ± 0.027	0.969 ± 0.012	0.920 ± 0.016	0.974 ± 0.012	0.975 ± 0.008
FlowMol2 (ref. 12)	0.959 ± 0.007	0.594 ± 0.009	0.944 ± 0.007	0.746 ± 0.010	0.938 ± 0.005	0.861 ± 0.012
Megalodon-flow^13^	0.990 ± 0.003	0.632 ± 0.011	0.987 ± 0.004	0.948 ± 0.003	0.958 ± 0.004^b^	0.949 ± 0.002^b^

a JODO was trained with the EQGAT-diff objective, using categorical diffusion instead of the original Gaussian formulation for categorical variables.

b Indicates results from a retrained “quick” variant, differing from the original paper which reported results for a larger model.

Correcting the numerical bug that erroneously rounded the contribution of aromatic bonds from 1.5 to 1 (without adjusting the lookup table) causes a dramatic drop in molecular stability. This can be observed by comparing the first two columns of Table 1. Additionally, this demonstrates that neither 1 nor 1.5 provides a universally reliable estimate for the contribution of an aromatic bond to atomic valency.

We propose two strategies to address the limitations in molecular stability computation. The first strategy involves enhancing the valency lookup table by explicitly accounting for aromaticity. Instead of the conventional tuples (element, formal charge, valency), we construct a more nuanced table indexed by (element, number of aromatic bonds, formal charge, valency), with the associated values representing allowed non-aromatic bond valencies—i.e., total bond order excluding contributions from aromatic bonds (see SI Table 5). In this formulation, each atom's bonding environment is described by the tuple (*n*_arom_, *v*_other_), where *n*_arom_ is the number of aromatic bonds and *v*_other_ is the total bond order from non-aromatic bonds. For example, a carbon atom in benzene typically exhibits configurations like (2, 1)—two aromatic bonds and one single bond—or (3, 0), as illustrated in Fig. 1. Remarkably, adopting this refined lookup table results in molecular stability scores only 1–3% lower than originally reported using flawed metrics (third column in Table 1). While modest, this deviation can meaningfully influence the comparative assessment of generative models and may introduce bias into subsequent benchmark studies if left uncorrected.

An alternative approach involves retraining models on a reprocessed dataset consisting exclusively of kekulized molecules, thereby completely removing ambiguity associated with aromaticity in valency computation. We prepared a revised version of the GEOM-drugs dataset so that all molecules were kekulized; there is no explicit modeling of aromatic bonds. As illustrated in Table 1, models trained on the kekulized dataset exhibited molecular stability comparable to previously published results when valencies were computed correctly. Notably, all models except Megalodon Flow demonstrated an average 5% improvement in validity. Megalodon Flow did not show similar improvements. We hypothesize that this discrepancy arises due to smaller neural network architecture used for Megalodon Flow, a decision necessitated by limited computational resources available for this study.

**Fig. 3:**
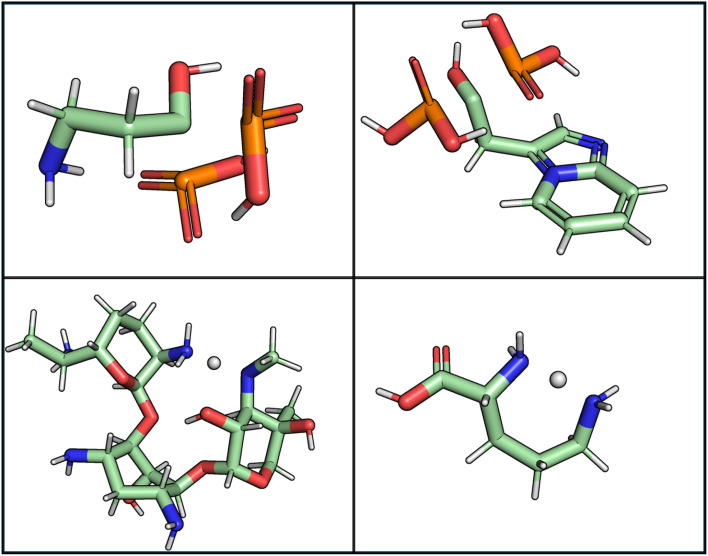
Examples from GEOM-drugs where GFN2-xTB failed and resulted in fractured molecules. The first row of molecules have neutral carbon with valency 2 and those in the second row have a positively charged hydrogen with valency zero.

We encountered another issue with GEOM-drugs: recomputing the valency table on the raw GEOM-drugs dataset revealed unusual valencies. Resulting from rare failure in the GFN2-xTB geometry optimization step used to produce the dataset. These failures produced fragmented molecules and unstable valencies such as hydrogen atom with no covalent bonds or neutral carbon with a valency of two. Examples of these instances are shown in Fig. 3. We removed molecules from GEOM-drugs that were fragmented into multiple disconnected components due to failed GFN2-xTB geometry optimization. This led to the exclusion of 0.18% of the dataset; although this is not enough data to significantly impact model performance, the presence of these molecules alters the resulting valency lookup table.

To summarize, neither treating aromatic bonds as contributing a valence of 1 nor 1.5 yields chemically accurate results. By correcting the valency table using a refined tuple representation, which captures the number of aromatic bonds separately, the resulting molecular stability scores decrease modestly by 1 to 3%. However, since most reported stability values exceed 0.9, even such small discrepancies can have an outsized influence, potentially skewing model development and encouraging optimization against a chemically flawed metric. Notably, retraining models on a reprocessed dataset with kekulized molecules, *i.e.*, without explicit aromatic bonds, leads to approximately a 5% improvement in validity for 4 of 6 evaluated models. Together, these results underscore the critical importance of chemically sound preprocessing and robust evaluation protocols in the development of 3D molecular generative models.

We make available in the attached github repository the filtered GEOM-drugs dataset with kekulized molecules, the scripts for producing the filtered dataset from the original GEOM dataset, and an implementation of the molecular stability metric that does not permit erroneous atomic valencies.

## 3D molecule evaluation

### Challenges in proper and accurate 3D structure assessment

Current 3D molecular generative models face significant challenges in evaluating the geometric quality of their outputs. In particular, models trained on the GEOM-drugs dataset often exhibit issues stemming from the evaluation protocols themselves.

A widely used approach involves defining a bond length lookup table and applying fixed thresholds to assess 3D molecular stability.^15–20^ However, this method is problematic for GEOM-drugs. Only 86.5% of atoms meet the specified atom–atom distances, and just 2.8% of molecules pass the stability criterion. The conformers in the GEOM-drugs dataset are optimized with respect to GFN2-xTB,^5^ a semi-empirical quantum chemical potential. Thus, the observed bond lengths reflect the GFN2-xTB energy landscape. Molecules obtained from other sources, such as the Cambridge Structural Database (CSD), may exhibit different geometries. This metric produces implausibly low stability rates yet remains widely adopted in new studies.

A more recent trend is to assess geometric quality by comparing distributions of bond lengths and angles using Wasserstein distance between generated and training data.^10,14,23^ This approach is more principled. However, distributional metrics are difficult to interpret, which makes it harder to extract chemically meaningful insights.

Other studies have proposed evaluating generated molecules by computing the relaxation energy using molecular mechanics force fields.^9,21,24^ A common choice has been the Merck Molecular Force Field (MMFF);^25^ however, this potential function differs substantially from GFN2-xTB,^5^ the potential used to produce the GEOM-drugs dataset. For conformers in the GEOM-drugs dataset, the mean relaxation energy difference Δ*E*_relax_ when re-optimized with GFN2-xTB is close to zero, as expected. In contrast, MMFF evaluation yields a mean Δ*E*_relax_ of. This is consistent with prior reports of MMFF errors in the 15–20 kcal mol^−1^ range relative to higher-level methods.^26^

As we will demonstrate, current state-of-the-art generative models produce structures that are closer to the GFN2-xTB ground truth on the GEOM-drugs dataset than their MMFF94-optimized counterparts. This renders MMFF-based comparisons unreliable and masks meaningful differences between models. However, MMFF energy can still serve as a coarse-grained filter to eliminate structurally implausible molecules, similar to its use in PoseBusters^27^ for energy-based outlier detection. Given the widespread reliance on inadequate metrics, we argue that a GFN2-xTB-based evaluation pipeline is necessary for accurately assessing the practical performance of 3D molecular generative models.

### GFN2-xTB energy-based geometry benchmark

GEOM-drugs geometries are optimized using the GFN2-xTB semi-empirical quantum calculation method. Therefore, it is essential to use the same method to assess the structural integrity of generated molecules. One approach is to measure of how close a generated structure is to the closest local minima of the given energy function. To measure this we suggest to assess differences in bond lengths, bond angles, and torsion angles of generated and optimized counterparts. These quantities provide clear and interpretable measure of generated molecules for both computer scientists and computational chemists.

### Bond length differences

For each bond in the molecule, we compute the difference in bond lengths between the initial (generated) and optimized (relaxed) structures. Let *r*^init^_*ij*_ and *r*^opt^_*ij*_ denote the distances between atoms *i* and *j* in the initial and optimized conformations, respectively. The bond length difference Δ*r*_*ij*_ is calculated as:Δ*r*_*ij*_ = |*r*^init^_*ij*_ − *r*^opt^_*ij*_|

The average difference is reported as a result.

### Bond angle differences

For each bond angle formed by three connected atoms *i*, *j*, and *k*, we calculate the angle difference between the initial and optimized structures. Let *θ*^init^_*ijk*_ and *θ*^opt^_*ijk*_ represent the bond angles at atom *j* in the initial and optimized conformations, respectively. The bond angle difference Δ*θ*_*ijk*_ is given by:Δ*θ*_*ijk*_ = min(|*θ*^init^_*ijk*_ − *θ*^opt^_*ijk*_|, 180° − |*θ*^init^_*ijk*_ − *θ*^opt^_*ijk*_|)

As with bond lengths, the average difference is reported as a result.

### Torsion angle differences

Torsion angles involve four connected atoms *i*, *j*, *k*, and *l*. We compute the difference in torsion angles between the initial and optimized structures using:Δ*ϕ*_*ijkl*_ = min(|*ϕ*^init^_*ijkl*_ − *ϕ*^opt^_*ijkl*_|, 360° − |*ϕ*^init^_*ijkl*_ − *ϕ*^opt^_*ijkl*_|)where *ϕ*^init^_*ijkl*_ and *ϕ*^opt^_*ijkl*_ are the dihedral angles in the initial and optimized conformations, respectively. This formula accounts for the periodicity of dihedral angles, ensuring the smallest possible difference is used.

The average difference is reported as a result.

## Results

We report results for EQGAT, Megalodon-quick, SemlaFlow, FlowMol2, and Megalodon-flow, including both the median and mean relaxation energy Δ*E*_relax_—the energy difference between the initial and GFN2-xTB-optimized structures—as well as structural displacement metrics discussed above (see Table 2). For each model, 5000 molecules were evaluated, and a randomly selected subset of 5000 molecules from GEOM-drugs was used for baseline comparisons. To compute confidence intervals, all metrics were calculated across five equal-sized splits of 1000 molecules each. In Table 2, the row labeled “MMFF → GFN2-xTB” quantifies geometric and energetic discrepancies between MMFF-optimized structures and their GFN2-xTB-optimized counterparts. These values highlight the structural divergence between force-field and semi-empirical optimization methods. Diffusion-based models already surpass MMFF in structural precision. Furthermore, we observe a consistent performance gap between flow-matching and diffusion-based models, even when the architecture is the same. This discrepancy has not been emphasized in prior literature. Conclusions drawn in prior works may have been influenced by the limited precision of their evaluation methodologies.

**Table 2:** Energy relaxation and geometric deviation metrics across generative models. Bond lengths (Å), angles (degrees), and energies (kcal mol^−1^) are reported for valid molecules only. Diffusion-based models use 500 steps; flow-matching models use 100 steps. Δ*E*_relax_ denotes the energy difference between the initial and GFN2-xTB-optimized structures (*i.e.*, the generative model's deviation from the reference energy landscape). Δ*E*^MMFF^_relax_ denotes the MMFF94 energy difference between the initial structure and the structure optimized with MMFF94

Model	Bond length (×10^−2^)	Bond angles	Torsions Δ*E*_relax_	Median Δ*E*_relax_	Mean Δ*E*^MMFF^_relax_	Mean
GEOM-drugs	0.00 ± 0.001	0.001 ± 0.001	0.01 ± 0.01	0.000 ± 0.0001	0.001 ± 0.001	16.4 ± 0.2
MMFF → GFN2-xTB	1.12 ± 0.01	1.22 ± 0.004	4.89 ± 0.10	9.84 ± 0.06	11.4 ± 0.2	0.00 ± 0.05
EQGAT-diff	1.00 ± 0.04	1.15 ± 0.03	8.58 ± 0.11	6.40 ± 0.20	11.1 ± 0.8	28.4 ± 1.2
JODO	0.77 ± 0.01	0.83 ± 0.00	6.01 ± 0.07	4.74 ± 0.15	7.04 ± 0.20	22.1 ± 0.2
Megalodon	0.66 ± 0.02	0.71 ± 0.01	5.58 ± 0.11	3.19 ± 0.12	5.76 ± 0.27	21.6 ± 0.3
SemlaFlow	3.10 ± 0.23	2.06 ± 0.17	6.05 ± 0.56	32.3 ± 3.3	91.0 ± 21.7	69.6 ± 9.2
FlowMol2	1.30 ± 0.04	1.62 ± 0.02	15.0 ± 0.3	17.9 ± 0.5	24.3 ± 0.8	39.4 ± 1.2
Megalodon-flow	2.30 ± 0.02	1.62 ± 0.02	5.58 ± 0.19	20.9 ± 0.8	46.9 ± 8.6	45.5 ± 2.0

## Conclusion

In this study, we revisited the GEOM-drugs benchmark for 3D molecular generative model and uncovered several issues in current evaluation pipelines. We demonstrated that widely-adopted stability metrics are affected by code errors, chemically inconsistent valency tables, and reliance on postprocessed molecules, leading to inflated measures of model performance. Furthermore, our findings suggest that energy evaluations based on MMFF may not be reliable to characterize model performance. The difference between MMFF and the energy landscape of the dataset is substantially larger than the difference between the energy landscape of generated molecules and their training data.

To address these limitations, we proposed a refined evaluation protocol incorporating chemically sound valency definitions and GFN2-xTB-based energy and geometry assessments. Our experiments demonstrate that these corrections impact reported performance while preserving the relative rankings of models. Conversely, a high-quality dataset (error-free structures, consistent features, trustworthy labels) and relevant metrics (*e.g.* appropriate choice of level of theory or realistic valency lookup table) provide a solid foundation that can markedly improve model performance. We hope that this study will raise awareness about importance of chemical structure curation and processing. We believe these improvements will foster more reliable, interpretable, and chemically meaningful progress in 3D molecular generative modeling. Our recommended evaluation methods and GEOM-drugs processing scripts are available at https://github.com/isayevlab/geom-drugs-3dgen-evaluation.

## Implementation

We release all scripts and evaluation tools used in this work at:

https://github.com/isayevlab/geom-drugs-3dgen-evaluation.

The repository provides:

• Preprocessing utilities to sanitize, kekulize, and filter molecules from the GEOM-drugs dataset based on the number of fragments.

• Valency validation scripts that compute atom-level and molecule-level stability using chemically accurate valency tables.

• Energy-based geometry evaluation tools that compute structural deviations (bond lengths, angles, torsions) and relaxation energies using GFN2-xTB optimization.

Full usage instructions, examples, and dependencies are provided in the repository README.

## Conflicts of interest

There are no conflicts to declare.

## Appendices

### Appendix I: valency lookup tables for stability evaluation

To support rigorous evaluation of 3D molecular generative models, we include here a collection of empirical valency tables derived from the GEOM-drugs dataset. These tables are used to define chemically plausible bonding patterns, detect invalid topologies, and serve as standardized references for assessing molecular stability in raw generated molecules.

### Table 3: allowed valencies

This table summarizes the allowed valencies (*i.e.*, number of bonds including hydrogens) observed in valid GEOM-drugs structures. It lists configurations by element and formal charge. These values are used as a reference for atom-level and molecule-level stability metrics.

**Table 3:** Valency configurations derived from the GEOM-drugs dataset, organized by element and formal charge. Each cell lists the allowed valencies (including implicit hydrogens) observed for a given formal charge

Element	Charge −2	Charge −1	Charge 0	Charge +1	Charge +2	Charge +3
H	—	—	1	—	—	—
B	—	4	3	—	—	—
C	—	3	4	3	—	—
N	1	2	3	4	—	—
O	—	1	2	3	—	—
F	—	—	1	—	—	—
Si	—	—	4	5	—	—
P	—	—	3, 5	4	—	—
S	—	1	2, 3, 6	3	4	2, 5
Cl	—	—	1	2	—	—
Br	—	—	1	2	—	—
I	—	—	1	2	3	—
Bi	—	—	3	—	5	—

### Table 4: legacy and invalid valencies

This table contains valencies found in earlier versions of generative model evaluation pipelines, which include chemically implausible or legacy entries due to preprocessing bugs or failed optimization. It is frequently used to benchmark the quality of generated molecules and identify invalid valency assignments. Many recent studies reference or reuse this table directly.

**Table 4:** Historically used but chemically implausible valency configurations by formal charge. This reference table has been widely used to assess molecular generative models. Values highlighted in bold represent known incorrect or unstable configurations; values highlighted in italic were missing from historical tables but are observed in the dataset

Element	Charge −2	Charge −1	Charge 0	Charge +1	Charge +2	Charge +3
H	—	**0**	1	**0**	—	—
B	—	*4*	3	—	—	—
C	—	3	**3**, 4	3	—	—
N	1	2	**2**, 3	**2**, **3**, 4	—	—
O	—	1	2	3	—	—
F	—	**0**	1	—	—	—
Al	—	—	3	—	—	—
Si	—	—	4	*5*	—	—
P	—	—	3, 5	4	—	—
S	—	*1*, **3**	2, 6	**2**, 3	4	5
Cl	—	—	1	2	—	—
Br	—	—	1	2	—	—
Se	—	—	2, 4, 6	—	—	—
I	—	—	1	2	*3*	—
Hg	—	—	1, 2	—	—	—
Bi	—	—	3	—	5	—

### Table 5: aromatic valency tuples

This table enumerates all observed combinations of aromatic and non-aromatic bonds per element and charge in the dataset. Each entry is represented as a tuple , where is the count of aromatic bonds and is the total bond order from non-aromatic bonds. These tuples capture valency patterns that are otherwise ambiguous under standard counting, especially in polyaromatic and heterocyclic systems.

**Table 5:** Allowed valency combinations by element and number of aromatic bonds. Each cell shows normal valencies for a given atom type and number of aromatic neighbours (row) and formal charge (column). “—” indicates no observed combinations

Element	# Aromatic	Charge −2	Charge −1	Charge 0	Charge +1	Charge +2	Charge +3
H	0	—	—	1	—	—	—
B	0	—	4	3	—	—	—
C	0	—	3	4	3	—	—
	2	—	1	2, 1	1	—	—
	3	—	0	0	0	—	—
N	0	1	2	3	4	—	—
	2	—	0	0, 1	0, 1, 2	—	—
	3	—	—	0	0	—	—
O	0	—	—	2	3	—	—
	2	—	—	0	—	—	—
F	0	—	—	1	—	—	—
Si	0	—	—	4	5	—	—
P	0	—	—	3, 5	4	—	—
S	0	—	1	2, 3, 6	3	4	2, 5
	2	—	—	0	0, 1	—	—
	3	—	—	—	0	—	—
Cl	0	—	—	1	2	—	—
Br	0	—	—	1	2	—	—
I	0	—	—	1	2	3	—
Bi	0	—	—	3	—	5	—

### Appendix II: examples of fractured compounds in GEOM-drugs

Together, these tables offer a robust and chemically grounded framework for interpreting stability metrics and ensuring consistency in the evaluation of 3D molecule generation pipelines. Table 4 in particular is widely used in existing benchmarking literature and reproduced here for completeness.

### Appendix III: convergence analysis of evaluation sample size

**Fig. 4:**
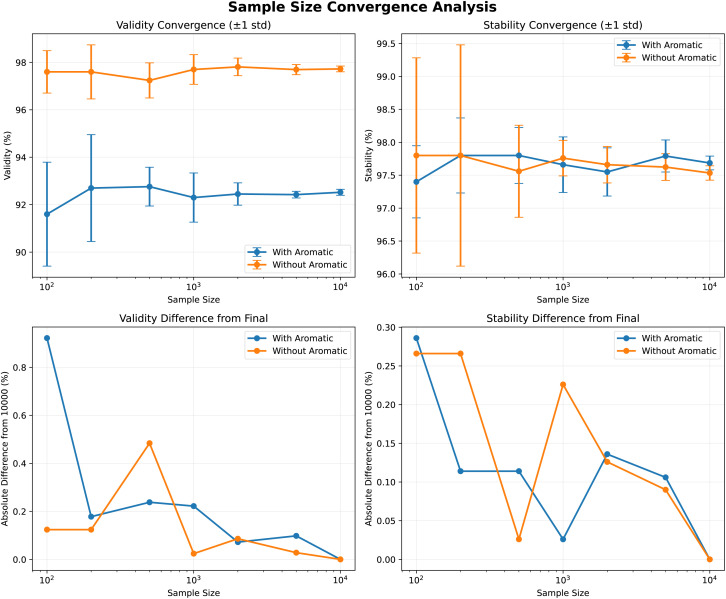
Mean and standard deviation of validity and stability metrics for the SemlaFlow model trained on kekulized *versus* explicit-aromatic GEOM-drugs molecules. Each point represents the mean across five independent 10k-molecule chunks; lines show cumulative subsampling at sizes from 1k to 10k per chunk. The std at 5k samples do not exceed 0.25%, demonstrating that 5k samples are sufficient to obtain stable estimates of evaluation metrics.

To verify that our evaluation sample size (5k molecules) is statistically adequate, we conducted a convergence analysis using SemlaFlow trained on both kekulized and explicit-aromatic variants of GEOM-drugs. As shown on Figure 4, we evaluated five independent 10k-molecule chunks and measured means and standard deviations for all metrics across cumulative subsamples. The differences in mean validity and stability metrics between 5k and 50k (5× 1k *vs.* 5× 10k) were ≤0.25%, well within the train-to-train variance observed for generative models. This confirms that using 5k samples provides representative metric estimates.

### Appendix IV: convergence analysis of evaluation sample size

**Fig. 5:**
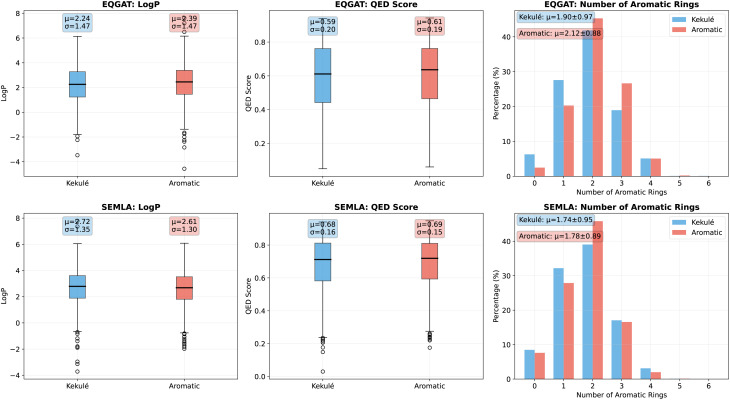
Comparative property distributions (log *P*, QED score, and aromatic ring counts) for molecules generated by EQGAT and SemlaFlow models trained on kekulized *versus* explicit-aromatic GEOM-drugs datasets. No significant shifts are observed across these properties, indicating that kekulization does not introduce bias in the learned chemical space representation.

We investigated whether kekulization biases downstream learning by comparing property distributions of molecules generated by EQGAT and SemlaFlow trained on Kekulized *versus* explicit-aromatic GEOM-drugs. As shown on Figure 5, we observed no significant distribution shifts for log *P*, QED score, or aromatic ring counts. This is consistent with the fact that RDKit’s aromaticity detection is a deterministic function of molecular topology—aromaticity can be algorithmically derived from connectivity and thus represents dependent rather than independent information. Given GEOM-drugs’ large scale, generative models have sufficient data to implicitly learn aromaticity patterns, showing that kekulization does not introduce meaningful bias.

## Data Availability

All data processing scripts, evaluation tools, and instructions for obtaining the corrected GEOM-drugs dataset used in this study are openly available at https://github.com/isayevlab/geom-drugs-3dgen-evaluation with the DOI https://doi.org/10.5281/zenodo.17089337. The repository includes code for molecule preprocessing (filtering, kekulization, valency table construction), valency-based stability evaluation, and GFN2-xTB–based energy and geometry benchmarking, enabling full reproduction of the reported results.
